# Evaluation of a simplified optimizer for MR‐guided adaptive RT in case of pancreatic cancer

**DOI:** 10.1002/acm2.12697

**Published:** 2019-08-24

**Authors:** Davide Cusumano, Luca Boldrini, Sebastiano Menna, Stefania Teodoli, Elisa Placidi, Giuditta Chiloiro, Lorenzo Placidi, Francesca Greco, Gerardina Stimato, Francesco Cellini, Vincenzo Valentini, Luigi Azario, Marco De Spirito

**Affiliations:** ^1^ Dipartimento di diagnostica per immagini, radioterapia oncologica ed ematologia Fondazione Policlinico Universitario “A. Gemelli” IRCCS Roma Italy; ^2^ Istituto di Radiologia Università Cattolica del Sacro Cuore Roma Italy; ^3^ Istituto di Fisica Università Cattolica del Sacro Cuore Roma Italy

**Keywords:** MR‐guided Radiotherapy, online adaptive radiotherapy, plan optimization, pancreatic cancer, IMRT

## Abstract

**Purpose:**

Magnetic resonance‐guided adaptive radiotherapy (MRgART) is considered a promising resource for pancreatic cancer, as it allows to online modify the dose distribution according to daily anatomy. This study aims to compare the dosimetric performance of a simplified optimizer implemented on a MR‐Linac treatment planning system (TPS) with those obtained using an advanced optimizer implemented on a conventional Linac.

**Methods:**

Twenty patients affected by locally advanced pancreatic cancer (LAPC) were considered. Gross tumor volume (GTV) and surrounding organ at risks (OARs) were contoured on the average 4DCT scan. Planning target volume was generated from GTV by adding an isotropic 3 mm margin and excluding overlap areas with OARs.

Treatment plans were generated by using the simple optimizer for the MR‐Linac in intensity‐modulated radiation therapy (IMRT) and the advanced optimizer for conventional Linac in IMRT and volumetric modulated arc therapy (VMAT) technique. Prescription dose was 40 Gy in five fractions. The dosimetric comparison was performed on target coverage, dosimetric indicators, and low dose diffusion.

**Results:**

The simplified optimizer of MR‐Linac generated clinically acceptable plans in 80% and optimal plans in 55% of cases. The number of clinically acceptable plans obtained using the advanced optimizer of the conventional Linac with IMRT was the same of MR‐Linac, but the percentage of optimal plans was higher (65%). Using the VMAT technique, it is possible to obtain clinically acceptable plan in 95% and optimal plans in 90% of cases.

The advanced optimizer combined with VMAT technique ensures higher target dose homogeneity and minor diffusion of low doses, but its actual optimization time is not suitable for MRgART.

**Conclusion:**

Simplified optimization solutions implemented in the MR‐Linac TPS allows to elaborate in most of cases treatment plans dosimetrically comparable with those obtained by using an advanced optimizer. A superior treatment plan quality is possible using the VMAT technique that could represent a breakthrough for the MRgART if the modern advancements will lead to shorter optimization times.

## 1 INTRODUCTION

To date, pancreatic cancer represents one of the most aggressive tumors with a 5 years overall survival (OS) rate ranging from 5% to 20%, depending on stage at diagnosis. Surgery still represents the most valuable treatment option, although only 20% of patients appears to be candidate for resection at diagnosis.^1^, ^2^

The large majority of these patients presents indeed unresectable locally advanced tumors, whose clinical management is much more complex and characterized by very poor prognosis. Different clinical trials have demonstrated that hypofractionated radiotherapy (RT) combined with chemotherapy may improve OS for patients affected by LAPC, even if surrounding organs at risk (OARs) toxicity still remains a strong dose limiting factor in this setting.^3^, ^4^

The recent development of hybrid magnetic resonance‐guided adaptive radiotherapy (MRgART), characterized by high soft‐tissue contrast images, allows for online modification of the dose distribution taking into account the daily positions of the treatment volumes, thus allowing to safely deliver high doses to the target and minimizing the dose to OARs.^5^, ^6^

To date, two MR‐Linac systems are available for clinical practice: MRIdian (ViewRay Inc, Mountain View, CA, USA) which couples a 0.35 T MRI scanner with a 6 MV Flattening Filter Free (FFF) Linac and Unity (Elekta, Stockholm, Sweden) which mounts a 1.5 T MRI scanner and a 7 MV FFF Linac.^7^, ^8^, ^9^

Both systems are based on a transverse geometry system, where the magnetic field (B) force lines are transversely oriented with respect to the radiation beam axis.

The innovative solutions offered by the hybrid systems allow the delivery of online adapted treatment plans: to this end the speed of plan optimization becomes crucial, as the patient is waiting on couch during the entire adaptive procedure (that can last up to 50 min) and unnecessary delays could cause anatomical changes, jeopardizing plan integrity or exhausting patient’s endurance in treatment position.^10^

The MRIdian Linac treatment planning system (TPS) to date offers two solutions to optimize the treatment plans. The most common version used during adaptive procedures implements a simplified optimization algorithm based on a convex nonlinear programming model that aims to build a simple cost function with one global minimum. Having calculation speed as priority, this optimizer allows to put in the cost function only two parameters for the target volume (minimum and maximum dose) and one maximum dose constraint for each considered OAR. No dose‐volume constraints can be put in this version of optimizer.^11^

A new version was recently introduced, that allows to consider more than one constraint for OARs and more than two constraints for the target volume. Although it offers also the opportunity to put in both dose constraints and dose objectives, its application during the adaptive procedure is still quite limited, as a quick solution of the cost function is not always reachable.

The recent introduction of these fast and simplified plan optimizers leads to the necessity to test their ability to elaborate dose distributions comparable with those created by traditional advanced optimizers, which can consider many dose to volume constraints in their cost function.

In this study, the RT treatment plans generated using the simplified optimizer of the MRIdian Linac TPS (version 4.5.1.239, ViewRay, Mountain View, CA, USA) were compared with those obtained using an advanced optimizer for traditional Linac plans (Eclipse® version 11.0, Varian, Palo Alto, CA, USA).

## 2 METHODS

### 2.1 Patients and treatment planning

Twenty consecutive patients affected by LAPC and treated in our institution from March 2017 to September 2018 were retrospectively enrolled in this study. A four‐dimensional computed tomography (4DCT) was acquired as simulation imaging and the average image was chosen to plan the radiation treatment.

Gross tumor volume (GTV), stomach, duodenum, liver, bowel bag, spinal cord, and kidneys were contoured by two radiation oncologists.

Clinical target volume (CTV) was considered equal to GTV and planning target volume (PTV) was generated from the CTV, through an isotropic 3 mm margin expansion and excluding any possible overlap with the adjacent OARs.

MRI Linac and traditional Linac treatment plans were then calculated on the same average 4DCT image, in order to test the performance of the two optimizers, working with the same treatment volumes.

The treatment plans with the advanced optimizer were generated considering as delivery unit a 6 MV FFF Linac (Edge Radiosurgery System, Varian, Palo Alto, CA, USA) with comparable physical characteristics to the MRIdian Linac, in order to avoid any bias due to different hardware.

The technical characteristics of the two systems are summarized in Table 1.

**Table 1:** Technical characteristics of the RT machines.

Technical characteristic	MRIdian linac	Varian edge
Beam	6 MV FFF	6 MV FFF
Dose rate	650 cGy/min	1400 cGy/min
Source to axis distance	90 cm	100 cm
Leaf width of multileaf collimator (MLC) at isocenter	0.415 cm	0.25 cm
Maximum field size	27 × 24 cm^2^	32 × 22 cm^2^
Minimum field size	0.41 × 0.2 cm^2^	0.25 × 0.2 cm^2^

The treatment plans were generated on both systems using the intensity‐modulated radiation therapy (IMRT) step and shoot technique with 20 equidistant angular beams. For the traditional Linac, the treatment plans were also optimized using the volumetric modulated arc therapy (VMAT) technique with dual arc.

No VMAT solutions are currently available on MRI Linac: the high number of IMRT beams was therefore chosen to mimic a VMAT dose distribution.

All treatment plans were performed by the same planner in order to avoid the inter‐operator variability bias. Prescribed dose was 40 Gy in 5 fractions to PTV for all the plans.

The dose distributions were calculated using the Analytical Anisotropic Algorithm for VMAT and IMRT traditional Linac plans and a Graphical Power Unit‐accelerated Montecarlo algorithm for the MR‐Linac plans.^12^

An isotropic grid size of 2 mm was set for dose calculation in both systems, as recommended by AAPM TG101 for SBRT treatment plans.^13^

The fluence map obtained during the optimization step on the MR‐Linac TPS was calculated using a spatial resolution of 2 mm and considering the presence of the magnetic field.

The MR‐Linac treatment plans were performed using the simplified version of the optimizer and considering a maximum number of 140 segments per plan and a total treatment time not superior to 25 min.

The Romeijn option that simultaneously calculate multiple plans with different number of segments, was turned off as it has a huge impact on the optimization time. Dose calculation was performed considering the presence of magnetic field and setting 2.4 million of histories, with a dose uncertainty equal to 1%.

The limit of 140 segments was kept constant also for the IMRT plans calculated on the conventional Linac, to ensure an equal comparison between the two technologies.

The mean time needed to optimize and calculate the different treatment plans was measured, excluding the time necessary to set the beam geometry and to create the supporting structures necessary to optimize the dose distribution, as they have been already created in a typical online adaptive scenario. The time necessary to obtain a first dose distribution was separated to that one required to reach the final solution.

All contours were double checked by two radiation oncologists on both planning systems, in order to reduce the uncertainties related to the different interpolation and smoothing algorithms adopted by the two TPSs.

### 2.2 Plan optimization strategy

The cases were classified in two different planning categories, considering the distance between the PTV outer margin and the closest OAR, in order to take into account the clinical heterogeneity observable in LAPC radiation treatment.

This classification has been applied considering the distance between the PTV outer margin and the closest OAR and setting a threshold of 3 mm.

OARs constraints had to be respected in all the cases and treatment plan optimization was stopped only when one organ at risk maximum constraint was reached.

#### 2.2.1 Planning category 1

In the cases where the closest OAR had a distance to the PTV inferior to 3 mm, the primary objective was to obtain a CTV coverage higher or equal to 99.5 % with the 95% of the prescription dose (CTV V95% ≥ 99.5%), ensuring in the meanwhile the D1% of CTV inferior to 50 Gy. The secondary objective was to obtain the highest PTV coverage respecting all the OARs dose constraints.

#### 2.2.2 Planning category 2

In the cases where the minimum distance between the PTV margin and the OARs was larger than 3 mm, the objectives of the planning category 1 were considered as preliminary conditions to be achieved. For these more anatomically advantageous cases the primary planning objective was to obtain a PTV coverage higher or equal to 98 % with the 95% of the prescription dose (PTV V95% ≥ 98%).

For both categories, very tight constraints in terms of target coverage were adopted, in order to ensure the achievement of a full optimization of the treatment plans.

All the plans were normalized setting the prescription dose at 50% of the PTV and allowing a D1% of PTV not higher than 50 Gy.

Table 2 reports the dose constraints considered for the OARs for pancreatic MRgART, as proposed by Bouhoudi and colleagues.^14^

**Table 2:** Dose constraints adopted for the optimization of the treatment plans.

Parameter	MR‐Linac		VMAT		IMRT	
	Mean value	σ	Mean value	σ	Mean value	σ
CTV						
V95 (%)	99.63	0.62	99.73	0.67	99.77	0.83
V105 (%)	0.34	0.50	0.00	0.00	0.00	0.00
D2% (Gy)	41.29	0.34	40.60	0.18	40.58	0.24
D98% (Gy)	38.90	0.44	39.25	0.51	39.31	0.57
TV						
V10Gy (cc)	823.99	567.85	778.02	520.32	699.93	577.55
V20Gy (cc)	204.30	154.02	214.57	153.89	181.01	152.00
V38Gy (cc)	58.64	50.86	57.45	48.66	57.20	50.15
PTV						
V95 (%)	97.76	1.96	98.54	1.53	98.62	1.69
V105 (%)	0.31	0.46	0.00	0.01	0.03	0.11
D2% (Gy)	41.26	0.35	40.66	0.18	40.77	0.23
D98% (Gy)	37.85	0.87	38.28	1.08	38.41	1.03
Duodenum						
V33Gy(cc)	0.42	0.43	0.31	0.39	0.36	0.43
V25Gy (cc)	1.90	2.12	2.07	2.51	2.50	3.38
Stomach						
V33Gy(cc)	0.20	0.33	0.14	0.36	0.18	0.33
V25Gy (cc)	1.52	2.10	2.11	4.45	1.88	3.53
Bowel bag						
V33Gy(cc)	0.16	0.28	0.15	0.55	0.09	0.25
V25Gy (cc)	1.47	2.37	0.71	1.89	0.94	2.52
Right kidney						
V12Gy (%)	3.75	7.16	2.28	4.74	2.45	3.76
Left kidney						
V12Gy (%)	3.37	6.16	0.83	2.47	1.69	4.33
Liver						
V12Gy (%)	4.44	5.54	3.78	4.81	4.07	5.05
Dosimeter indicators						
CI (PTV)	1.20	0.08	1.23	0.10	1.17	0.11
HI (CTV)	0.07	0.04	0.03	0.01	0.03	0.02
HI (PTV)	0.09	0.03	0.06	0.03	0.06	0.03
GI	4.27	0.99	4.69	1.21	3.81	0.88

### 2.3 Plan evaluation

All plans were firstly evaluated by two radiation oncologists to assess their clinical acceptability with the following criteria: 95% of the PTV had to be covered with at least 95% of the prescription dose and OARs constraints had to be successfully met.^15^

In particular, considering the two planning categories defined, a treatment plan was indicated as “clinically acceptable” if the V95% of CTV was higher or equal to 99.5% and the V95% of PTV was comprised between 95% and 98%. A plan was labeled as “optimal” if two conditions were simultaneously reached: CTV V95% ≥ 99.5% and PTV V95% ≥ 98%. All the cases where CTV V95% was < 99.5% or PTV V95% was < 95% were considered as not clinically acceptable plans.

The overall dosimetric comparison of the dose distributions obtained from the two systems was based on target coverage, OARs sparing and low dose irradiated volumes.

The results were compared using the Wilcoxon signed‐rank test and differences were considered statistically significant for *P*‐value lower than 0.05.^16^

Three dosimetric indicators were adopted to evaluate the quality of the dose distributions.

The conformity index (CI) was used to evaluate the conformity of the dose distribution on the PTV^15^:

$$\text{CI}=\frac{V38\;\text{Gy}}{V\left(\text{PTV}\right)}$$

where V38Gy is the volume of the 95% of the prescription dose (38 Gy), while V(PTV) is the volume of the target structure.

The homogeneity index was calculated for CTV and PTV as defined by ICRU 83:

$$\text{HI}=\frac{D2\%\left(\mathrm{Target}\right)-D98\%\left(\mathrm{Target}\right)}{D50\%\left(\mathrm{Target}\right)}$$

where D2%, D98% and D50% represent the absolute doses covering the 2%, the 98%, and the 50% of the target structures, respectively.

The low‐dose spread was evaluated considering the volumes in cubic centimeters (cc) of the 10 and 20 Gy doses and in terms of gradient index (GI)^17^:

$$\text{GI}=\frac{V_{50\%\;of\;the\;prescription\;dose}}{V_{100\%\;of\;the\;prescription\;dose}}$$

## 3 RESULTS

**Figure 1:**
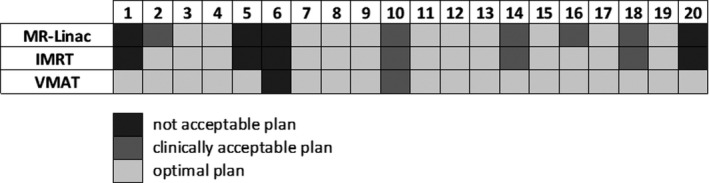
Schematic representation of the results obtained in terms of clinical evaluation for the MR‐Linac in IMRT modality (MR‐Linac) and the traditional Linac in IMRT (IMRT) and VMAT (VMAT) modality. A plan was considered as “clinically acceptable” if the V95% of CTV ≥ 99.5% and V95% of PTV was comprised between 95% and 98%. A plan was labeled as “optimal” if CTV V95% ≥ 99.5% and PTV V95% ≥ 98%. All the other cases were considered as not clinically acceptable plans.

For nine cases analyzed the minimum distance between the PTV margin and the closest OAR was smaller than 3 mm. A schematic representation of the results obtained for the clinical evaluation of the treatment plans is reported in Fig. 1.

The simplified optimizer of the MR‐Linac generated clinically acceptable plans in 80% of cases (16/20) and optimal plans in 55% of cases (11/20).

The number of clinically acceptable plans obtained using the advanced optimizer of the conventional Linac with IMRT technique was the same as that one obtained with the MR‐Linac, but the percentage of optimal plans (65%) was higher.

Using the traditional Linac with the VMAT technique only in one case out 20 the treatment plan generated did not reached the clinical acceptability and in 90% of cases optimal plans were generated.

For patient 6 all the approaches failed in calculating a clinically acceptable plan, as the tumor and critical structures geometry resulted to be unfavorable for SBRT delivery.

The mean time required to obtain an initial dose distribution that solve the cost function was 1.6 ± 0.5 min for the MR‐Linac, 1.8 ± 0.7 min for the IMRT with traditional Linac and 8.1 ± 1.8 min for the VMAT Linac.

The mean time to obtain the dose distribution that meets all the dose constraint was: 5.6 ± 1.7 min for the MR‐Linac, 5.4 ± 2.1 min for the IMRT with traditional Linac and 13.6 ± 2.9 min for the VMAT Linac.

Table 3 reports the mean values and the relative standard deviations for all the investigated dose metrics parameters.

**Table 3:** Mean values and relative standard deviations calculated for the different dosimetric indicators in the case of MR‐Linac, IMRT, and VMAT traditional Linac.

Parameter	MR‐Linac		VMAT		IMRT	
	Mean value	σ	Mean value	σ	Mean value	σ
CTV						
V95 (%)	99.63	0.62	99.73	0.67	99.77	0.83
V105 (%)	0.34	0.50	0.00	0.00	0.00	0.00
D2% (Gy)	41.29	0.34	40.60	0.18	40.58	0.24
D98% (Gy)	38.90	0.44	39.25	0.51	39.31	0.57
TV						
V10Gy (cc)	823.99	567.85	778.02	520.32	699.93	577.55
V20Gy (cc)	204.30	154.02	214.57	153.89	181.01	152.00
V38Gy (cc)	58.64	50.86	57.45	48.66	57.20	50.15
PTV						
V95 (%)	97.76	1.96	98.54	1.53	98.62	1.69
V105 (%)	0.31	0.46	0.00	0.01	0.03	0.11
D2% (Gy)	41.26	0.35	40.66	0.18	40.77	0.23
D98% (Gy)	37.85	0.87	38.28	1.08	38.41	1.03
Duodenum						
V33Gy (cc)	0.42	0.43	0.31	0.39	0.36	0.43
V25Gy (cc)	1.90	2.12	2.07	2.51	2.50	3.38
Stomach						
V33Gy (cc)	0.20	0.33	0.14	0.36	0.18	0.33
V25Gy (cc)	1.52	2.10	2.11	4.45	1.88	3.53
Bowel bag						
V33Gy (cc)	0.16	0.28	0.15	0.55	0.09	0.25
V25Gy (cc)	1.47	2.37	0.71	1.89	0.94	2.52
Right kidney						
V12Gy (%)	3.75	7.16	2.28	4.74	2.45	3.76
Left kidney						
V12Gy (%)	3.37	6.16	0.83	2.47	1.69	4.33
Liver						
V12Gy (%)	4.44	5.54	3.78	4.81	4.07	5.05
Dosimeter indicators						
CI (PTV)	1.20	0.08	1.23	0.10	1.17	0.11
HI (CTV)	0.07	0.04	0.03	0.01	0.03	0.02
HI (PTV)	0.09	0.03	0.06	0.03	0.06	0.03
GI	4.27	0.99	4.69	1.21	3.81	0.88

**Figure 2:**
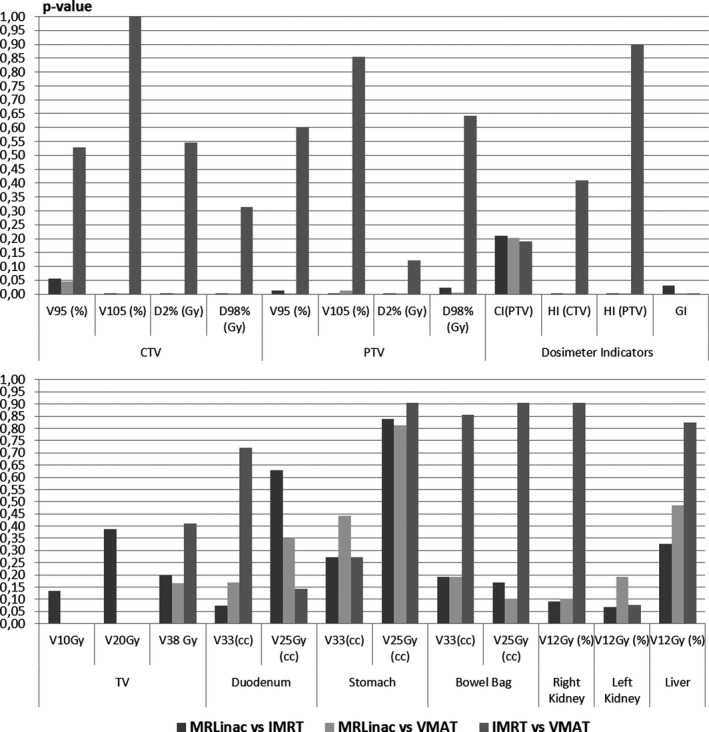
Results in terms of *P*‐values for Wilcoxon signed‐rank test obtained for target structures and dosimetric indicators (upper part) and for OARs and treated volumes (lower part). Comparisons were performed between MR‐Linac and IMRT plans obtained with traditional Linac, MR‐Linac, and VMAT plans on traditional Linac, and between the two delivery modalities of traditional Linac.

Fig. 2 shows the *P*‐values of the WMW test obtained comparing the parameters obtained in the MR‐Linac plans with those obtained considering the traditional Linac with IMRT and VMAT technique.

The traditional Linac plans generated using the advanced optimizer, both for IMRT and VMAT technique, showed higher dose homogeneity on the target structures respect to those obtained using the MR‐Linac.

In particular, the values of HI, D2% and V105 for CTV and PTV were significantly lower using the traditional Linac, while the D98% of CTV and the V95 of CTV and PTV resulted to be significantly higher.

**Figure 3:**
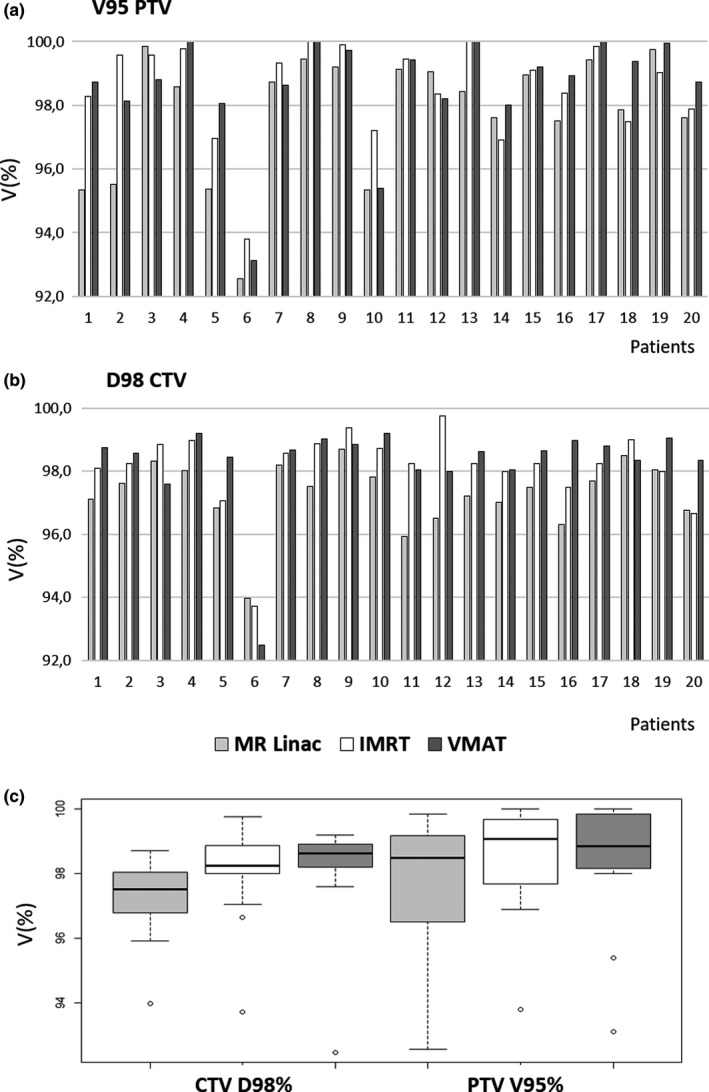
Bar plot (a and b) and box plot (c) showing the values of target coverage expressed as D98% CTV and V95% PTV for MR‐Linac, IMRT and VMAT for conventional Linac.

Fig. 3 shows the results obtained in terms of target coverage expressed as V95% PTV and D98% CTV. The comparison in terms of D98% of CTV was statistically significant between MR‐Linac and IMRT (*P* < 0.001) and between MR‐Linac and VMAT (*P* = 0.001) but not between IMRT and VMAT (*P* = 0.546). Similar behavior was founded for V95% of PTV (*P* = 0.012 between MR‐Linac and IMRT, *P* = 0.003 between MR‐Linac and VMAT, *P* = 0.601 between IMRT and VMAT).

Population mean DVH curves were also reported in Fig. S1 of supplementary materials.^18^

Regarding the analysis on the dosimeter indicators, the HI values calculated on CTV and PTV for traditional Linac plans resulted to be significantly lower than those obtained on the MR‐Linac plans (*P* < 0.001 between MR‐Linac and VMAT and between MR‐Linac and IMRT both for CTV and PTV).

Similar behavior was founded for the gradient index (*P* = 0.029 between MR‐Linac and IMRT, *P* = 0.003 between MR‐Linac and VMAT, *P* < 0.001 between IMRT and VMAT).

**Figure 4:**
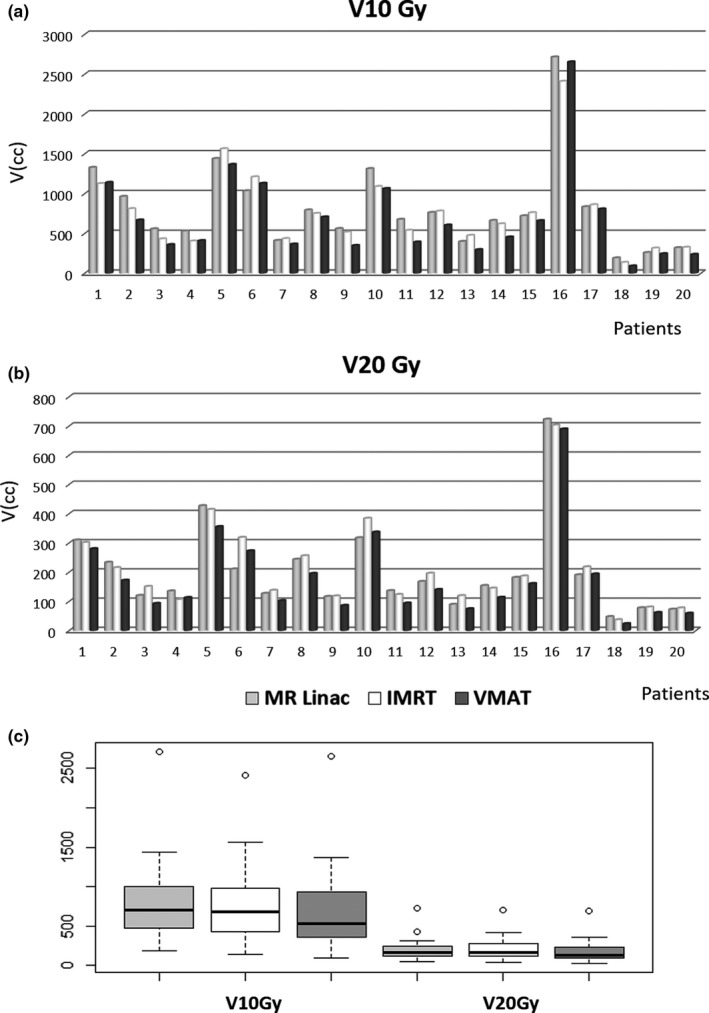
Bar plot (a and b) and box plot (c) showing low dose diffusion expressed as cubic centimeters of V20Gy and V10Gy for IMRT MR‐Linac, IMRT and VMAT and conventional Linac.

Statistical significance was also founded for the low dose values (V10 Gy and V20 Gy) in favor of the VMAT technique respect to the IMRT techniques (*P* = between MR‐Linac and VMAT, *P* = between IMRT and VMAT). Fig. 4 reports the volumes of 10 and 20 Gy for all the cases analyzed and the box plot analysis.

**Figure 5:**
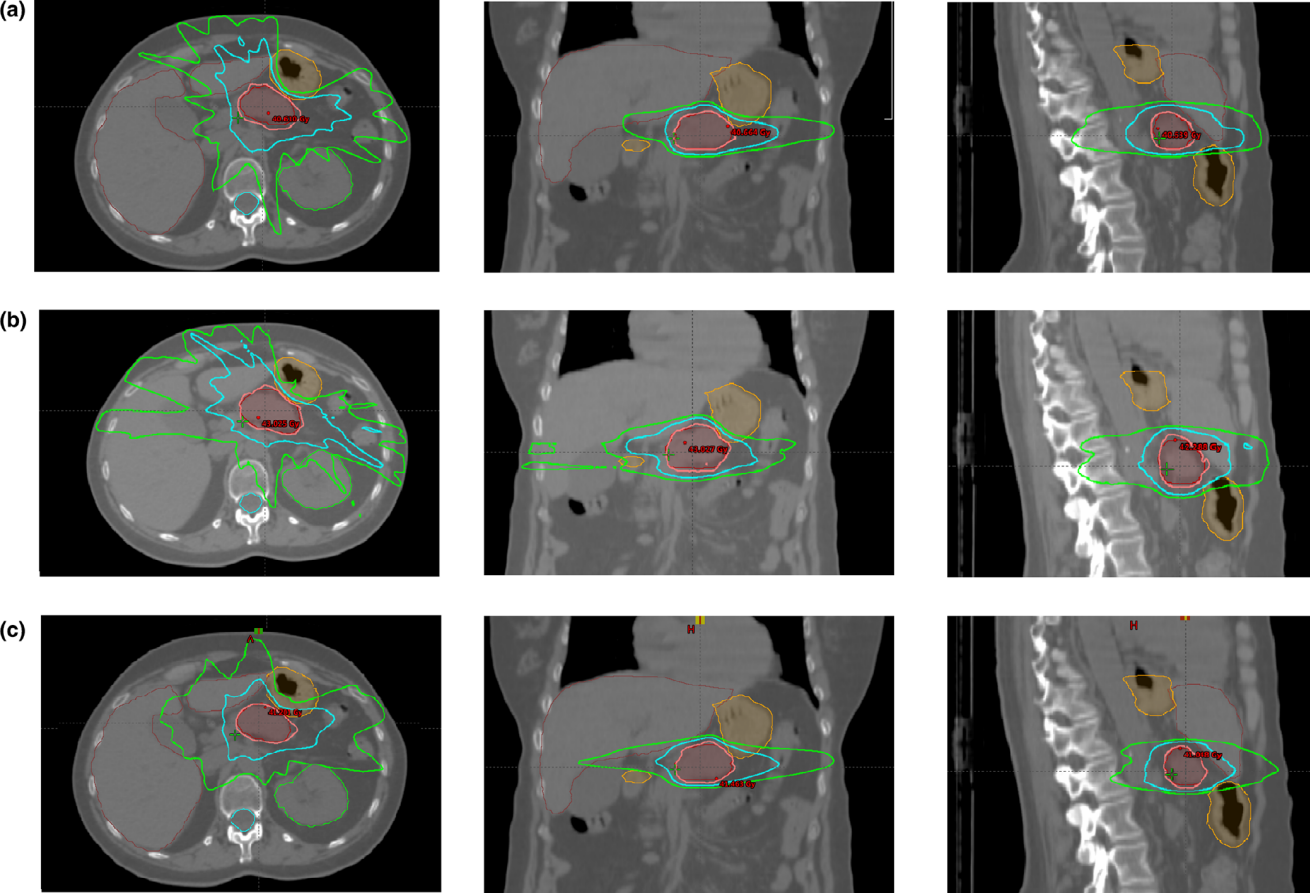
Example of dose distribution for a patient in the case of IMRT with conventional Linac (a), IMRT with MR‐Linac (b), and VMAT with conventional Linac (c). The pink isodose is the 95% of prescription dose (38 Gy), the cyan line represents the 20 Gy isodose, the green line is the 10 Gy isodose.

No significant difference was founded between MR‐Linac and traditional Linac with IMRT on the low‐dose spread (*P* = 0.133 for V10 Gy and *P* = 0.388 for V20 Gy).

The volumes of V10 Gy and V20 Gy were significantly lower for VMAT respect the two IMRT plans (*P* < 0.001 and *P* = 0.002 for V10Gy and V20Gy comparing VMAT and MR‐Linac; *P* = 0.001 and *P* < 0.001 between VMAT and IMRT with traditional Linac).

Lastly, no significant difference was observed among the different techniques for the OARs.

Fig. 5 shows the isodose lines obtained in a case analyzed with MR‐Linac and traditional Linac considering IMRT and VMAT techniques. A lower spread of low doses is visible using the VMAT technique.

## 4 DISCUSSION

Several studies have recently investigated the feasibility of generating clinically acceptable treatment plans for hybrid MRI guided treatment machines in different cancer sites, such as rectum, lung, and brain.^19^, ^20^

In particular, Ramey *et al*. recently compared the RT treatment plans calculated using two low tesla MRI‐guided RT systems (three Cobalt‐60 sources and MRIdian Linac version) with those obtained using a conventional Linac in the case of LAPC patients.^21^

Their conclusions asserted that only the MR‐Linac system is able to deliver treatment plans comparable with those calculated using a standard Linac, while the Cobalt‐60 version, appeared not able to reach the same treatment quality, mainly due to the low beam energy and the large width of multileaf collimator (MLC) leaves.

However, the comparison discussed in this study suffers from various limitations: it does not specify the technical characteristics of the standard Linac used; employs different dosimetrists to calculate the treatment plans; and it does not provide any information related to the analysis of low dose volumes. Furthermore, the lack of a predefined precise planning strategy (i.e., reference dose coverage values and fixed planning categories) does not allow to fully appreciate the quality of the plans and to understand if the best possible optimization limit had been reached.

The results reported in Fig. 1 show that the MR‐Linac and the traditional Linac with IMRT technique allow to deliver stereotactic dose values (40 Gy in five fractions) in 80% of clinical situations, ensuring high target coverage and respecting the OARs dose constraints. The VMAT technique allows instead to generate clinically acceptable plans in 95% of cases.

Based on these results, the implementation of the VMAT delivery technique in the MRgRT systems could offer SBRT treatment plans of a superior quality, allowing to treat most of the patients showing unfavorable anatomical situations.

The VMAT technique could become an optimal solution for online adaptive radiotherapy only if supported by an advanced optimizer with faster optimization time, as the current version requires too long time to provide a clinically acceptable solution.

Plans calculated with standard Linac, independent of the delivery technique, show higher dose homogeneity in comparison to MR‐Linac, maybe as direct consequence of the simplified optimizer adopted on the MR‐Linac TPS.

The IMRT plans shows also significantly larger values for V10Gy and V20 Gy in comparison to VMAT plans. Considering that no statistical significance was found for these parameters between MR‐Linac and IMRT traditional Linac plans, the low‐dose spread seems to be related to the delivery technique and not to the optimizer.

Time still remains the limiting factor for the VMAT implementation in the online adaptive procedures. Alternative approaches have been recently developed for delivery systems whose optimization algorithms do not allow adaptive online replanning procedures. The most common strategy consist of creating offline treatment plans libraries in which different possible scenarios are collected (e.g., different filling of hollow organs at risk), allowing the user to choose online the plan that fits the best with the anatomy of the day.^22^, ^23^

Taking into account the potentialities and the pitfalls of the described planning strategies, the results of this study show that the simplified optimizer implemented by the MRIdian Linac TPS allows for on‐line clinically acceptable dose distributions for MRgART in case of LAPC patients.

It should be also observed that this study was carried out considering equal therapy volumes, but the high soft‐tissue contrast offered by the possibility of having an on‐board MR scanner allows for a more precise contouring of the target and OARs on the MR‐Linac, and a consequent reduction of the PTV margin, not always achievable using the CT imaging.

MR imaging balances the observed disadvantage of larger low‐dose spread from the current MR‐Linac IMRT plans with the simple optimizer and motivates further development of target dose escalation clinical protocols, while possibly maintaining or reducing OAR toxicities, thanks to the possibly of daily plan adaptation based on the clinical situation.

## 5 CONCLUSIONS

Pancreatic cancer SBRT represents a challenge for RT delivery technologies for several reasons, such as the proximity between the tumor and radiosensitive OAR, and the significant inter‐ and intrafraction anatomical variability (e.g., different organ filling of hollow organs, breathing induced organ motion).

The MR‐Linac system, thanks to the high soft‐tissue contrast provided by the MRI and the possibility to adapt and gate the treatment by using real‐time cine MRI, appears to be a good system to address these sources of variability.

This study demonstrates that the fast‐simple optimizer implemented in the MR‐Linac TPS allows to elaborate IMRT treatment plans with dosimetric performances comparable to those obtained by using a traditional Linac with an advanced optimizer and same delivery technique. A superior treatment plan quality is possible using the VMAT technique that could represent a breakthrough for the MR‐guided Radiotherapy if the modern advancements will lead to shorter optimization times.

These results open new frontiers towards clinical approaches that aim to escalate the dose on target volumes while effectively sparing the surrounding healthy tissues.

## CONFLICT OF INTEREST

The authors have no relevant conflict of interest to declare.

## Supporting information


**Fig. S1.** DVH of average population for PTV (upper) and GTV (lower) in case of MR‐Linac, IMRT and VMAT of conventional Linac
