# Supplementary figures and images for: Evaluation of a simplified optimizer for MR‐guided adaptive RT in case of pancreatic cancer
Source: J Appl Clin Med Phys. 2019 Aug 24;20(9):20–30. doi: 10.1002/acm2.12697 (PMC6753732; doi:10.1002/acm2.12697)

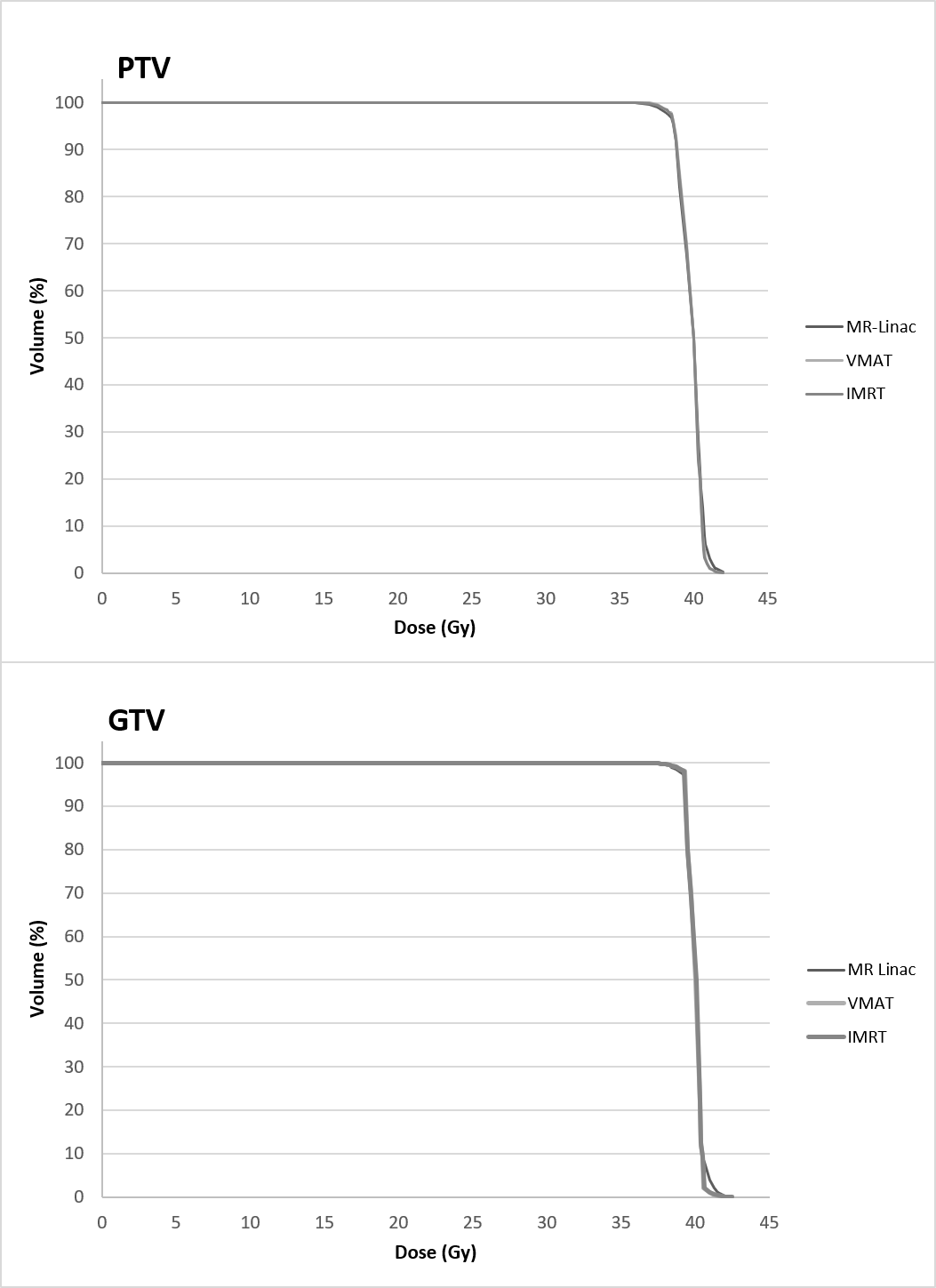

Supplement: Supplementary file 1 — Fig. S1. DVH of average population for PTV (upper) and GTV (lower) in case of MR‐Linac, IMRT and VMAT of conventional Linac [file ACM2-20-20-s001.tif]
